# Mapping sociodemographic and geographical differences in human papillomavirus non-vaccination among young girls in Sweden

**DOI:** 10.1177/14034948221075410

**Published:** 2022-02-04

**Authors:** Maria Wemrell, Raquel Perez Vicente, Juan Merlo

**Affiliations:** 1 Unit for Social Epidemiology, Department of Clinical Sciences, Lund University, Sweden; 2 Department of Gender Studies, Lund University, Sweden; 3 Centre for Primary Health Care Research, Region Skåne, Sweden

**Keywords:** HPV vaccination, non-vaccination, sociodemographic inequalities, Sweden, AIHDA

## Abstract

**Aims:**

Human papillomavirus (HPV) vaccination plays a key role in the prevention of cervical cancer. Yet, disparities in HPV vaccination in Sweden have persisted. Previous research on such disparities has typically focused on singular sociodemographic variables and measures of average risk. Using a multi-categorical approach and drawing on intersectionality theory, this study aimed to provide a more precise mapping of HPV non-vaccination among girls in different sociodemographic groups and geographical areas in Sweden during 2013–2020.

**Methods:**

Using nationwide register data, we conducted a multi-categorical analysis of individual heterogeneity and discriminatory accuracy complemented by a multilevel geographical analysis. We mapped HPV non-vaccination prevalence across 54 strata defined by parental income, education and country of birth, and urban versus rural place of residence. We also disentangled municipal and regional influences on HPV non-vaccination.

**Results:**

HPV non-vaccination was more common in groups with a low income, a low education and an immigration background, whereas among those with an immigration background, the association between income, education and HPV non-vaccination was more complex. Geographical differences were found between municipalities. However, the discriminatory accuracy of the sociodemographic and geographical groups was weak, and 50% of the non-vaccination cases were observed in eight strata, of which some are among those with low risk.

**Conclusions:**

**Our findings underscore the importance of universal yet tailored approaches, including providing adequate information about HPV vaccination in Swedish and other languages, and of health-care professionals displaying sensitivity to patients’ and parents’ questions or needs.**

## Introduction

In order to prevent human papillomavirus (HPV) infections and cervical cancer [1], in 2010, Sweden introduced a school-based, free-of-charge quadrivalent HPV vaccination programme for girls. Since then, HPV vaccination has been shown to reduce the population-level risk of invasive cervical cancer substantially [2]. In 2020, boys were included in the programme.

While HPV vaccine coverage varies between countries [3], in Sweden, it is comparatively high at around 80% [4]. The national goal of 90% coverage has not been reached, however. Alongside efforts towards improved understanding of attitudes and decision-making processes surrounding the HPV vaccine [5–7], including concerns about vaccine hesitancy [5,8], research has pointed to socio-economic differences in HPV vaccine uptake [9,10]. While in Sweden disparities have largely been augmented through the school-based HPV vaccination programme, differences pertaining to income, education and country of birth have persisted [9]. This is of particular concern, since low socio-economic position and immigration status are associated with a higher risk of non-attendance to cervical screening [11,12] and of incidence and mortality in cervical cancer, although the risk of cervical cancer is lower in some immigrant groups [13,14]. Geographical differences in HPV vaccination in Sweden have also been documented [4].

Still, studies of disparities in HPV vaccination uptake in Sweden are relatively sparse and mainly investigate the effects of singular socio-economic or geographical dimensions [9]. This may oversee differences between multidimensional socio-economic strata and at different geographical levels, discernible through multilevel and multi-categorical analyses. Therefore, this study aimed to provide an improved understanding of how combined sociodemographic and geographical dimensions affect HPV vaccination uptake in Sweden.

Our study draws on intersectionality theory [15], which is increasingly being used in population health research [16], as it enables an understanding of how combined socio-economic dimensions affect the outcome of interest. Intersectionality theory builds on the fundamental insight that different axes of social differentiation, including sex/gender, country of birth/racialisation and income/class, should not be understood as separate but as interwoven. Noted potential contributions of an intersectional perspective to social epidemiology include an increased specificity in the mapping of health inequalities through providing information about multiple strata defined by combinations of demographic and socio-economic dimensions (i.e. variables) [16]. Moreover, an intersectional perspective promotes the direction of focus towards societal structures and dynamics giving rise to health disparities [16].

We applied an analysis of individual heterogeneity and discriminatory accuracy (AIHDA), which is suitable for the multi-categorical study of health disparities [17], and complemented it with a geographical multilevel analysis [18,19] to disentangle the influence of municipalities and regions on HPV non-vaccination. Measures of discriminatory accuracy (DA) provide information about the ability of the categorisation at hand to distinguish between individuals with and without the outcome, depending on the presence of individual heterogeneity within groups. Such assessment can mitigate simplification or essentialisation of differences between groups and stigmatisation of groups with higher average risks. It may also prevent false expectations in low-risk groups and ineffective interventions due to over- or undertreatment [19].

### Aim

Using multi-categorical AIHDA, complemented by a multilevel geographical analysis, we aimed to provide an improved mapping of the sociodemographic and geographic distribution of HPV vaccine uptake in Sweden.

## Methods

### Study population

After approval by the Swedish Ethical Review Authority (no. 2020-05688), the National Vaccination Register (NVR) administered by the Public Health Agency was linked to the Register of the Total Swedish Population (TPR) and the Longitudinal Integration Database for Health Insurance and Labour Market Studies (LISA), which provides demographic and socio-economic information. The latter two are administered by Statistics Sweden, who performed the record linkage.

Our study population consisted of all girls between two and seven years of age living in Sweden on 31 December 2010 (*N*=315,652). Each age group was followed up during the period in which they were 10–12 years old (i.e. those who were seven years old in 2010 were followed up in 2013–2015, etc). We excluded those who died (*n*=95) or emigrated (*n*=1134) during the follow-up period and those with missing information (on parental education, *n*=2767). The final study population consisted of 311,656 girls (98.7% of the original sample).

### Assessment of variables

Following the school-based HPV vaccination programme, all girls are offered the HPV vaccination in the fifth school year, when they are 10–12 years old. Our outcome variable assessed whether the included girls received at least one vaccination in time (yes vs. no).

We computed a cumulative measure of individualised equivalised disposable family income by using information on absolute income for the years 2000, 2005 and 2010. For each of the three years, incomes were categorised into 25 groups by quantiles using the complete Swedish population. These groups were summed up, assigning to each individual a value between 3 (always in the lowest income group) and 75 (always in the highest income group). This cumulative income measure was divided into low, medium or high income by tertiles.

The parental educational achievement variable distinguished between girls who had, or had not, at least one parent with a tertiary education (i.e. high vs. low education).

The parental country of birth variable was categorised into native, mixed or immigrant based on whether both, only one or neither of the girls’ parents were born in Sweden. Parents with missing information were considered as immigrants, as all those born in Sweden are registered as such.

Place of residence was based on the location where the vaccination was administered, according to categories provided by Statistics Sweden (1–9) as big city (1–3), small city (4–5) and rural (6–9).

The multi-categorical variable was constructed through all possible combinations of the explanatory variables (3×2×3×3), forming 54 multi-categorical strata. Girls whose parents had a high income and education and were born in Sweden, living in a big city, were used as the reference stratum in the analyses.

In the multilevel analysis, we identified the municipality and county where the vaccination was administered.

### Statistical analyses

#### Multi-categorical and geographical analyses

Following a stepwise analytical approach described previously [18], we performed an AIHDA [17], which considers measures of average risk alongside measures of variance and DA. We first performed a logistic regression modelling HPV non-vaccination as a function of individual socio-economic variables (Model 1). Thereafter, we constructed a model (Model 2) including the same information but using the multi-categorical variable. The purpose of this second model was to provide a detailed mapping of HPV non-vaccination across the 54 strata. In a final step, Model 1 was expanded using geographical information consisting of random effects for the county and municipality levels (Model 3). This multilevel analysis provided information about geographical differences in HPV non-vaccination, adjusted for the socio-economic variables.

#### Measures of average risk

Associations were expressed as odds ratios (ORs). We also computed stratum-specific prevalence rates or absolute risks (ARs). We calculated 99% confidence intervals (CIs) to minimise the problem of multiple comparisons.

#### Assessment of DA

We assessed the DA of the regression models, that is, the predictive accuracy or the ability of the categorisations used in the models to distinguish between individuals who received HPV vaccination or not, by computing the area under the receiver operator characteristics curve (AUC) [18]. The curve was obtained by plotting the true-positive fraction against the false-positive fraction for binary classification thresholds of predicted risk. The AUC values range from 0.5 to 1, where 1 represents perfect discrimination and 0.5 indicates an absence of predictive accuracy. The DA can be classified as absent or very weak (AUC=0.5–0.6), weak (AUC >0.6–⩽0.7), strong (AUC >0.7–⩽0.8) or very strong (AUC >0.8) [20]. A weak DA may result from the existence of many false-positives, in this case of many non-vaccinated people in low-risk strata.

We calculated the incremental change in the AUC value (Δ-AUC), which quantifies improvements in DA yielded by a model compared to a previous one. If any statistical interaction of effects were identified in the multi-categorical variable, the AUC of Model 2 would be higher than that of Model 1 and the Δ-AUC>0 [17]. In Model 3, an Δ-AUC>0 would suggest the existence of a general contextual effect on HPV non-vaccination risk over and above the individual sociodemographic variables. In Model 3, we also calculated the intraclass correlation (ICC), which measured the share of the total individual variance in the latent propensity of HPV non-vaccination that existed at the municipality and county levels. The ICC is also a measure of DA [21].

IBM SPSS Statistics for Windows v22 (IBM Corp., Armonk, NY) for PC and MLwiN v3.00 called from within Stata v14.1 (StataCorp, College Station, TX) were used to conduct the analyses. The multilevel estimations were performed using Markov chain Monte Carlo methods.

## Results

Overall, as shown in Table I, 18.8% of the population did not receive any HPV vaccination on time. The probability of HPV non-vaccination was, on average, somewhat higher among girls whose parents had a low rather than a high education, and this probability increased as parental income decreased (Tables I and II). Non-vaccination was more common among girls with one or two parents born outside of Sweden compared to girls with parents born in Sweden, and among girls living in rural areas compared to those in big cities.

**Table I.** Prevalence of HPV non-vaccination non-receipt during 2013–2020, among 311,656 girls living in Sweden in 2010, according to income, parental education, parental country of birth and place of residence.

		**HPV non-vaccination, *n* (%)**
Total		58,640 (18.8)
Income	Low	28,702 (49.0)
	Medium	18,444 (31.5)
	High	11,494 (19.6)
Parental education	Low	33,760 (57.6)
	High	24,880 (42.4)
Parental country of birth	Native	35,683 (60.9)
	Mixed	9041 (15.4)
	Immigrant	13,916 (23.7)
Place of residence	Big cities	36,176 (61.7)
	Small cities	15,416 (26.3)
	Rural	7048 (12.0)

Figures are number (percentages).

HPV: human papillomavirus.

The multi-categorical analysis (Table II, Model 2) yielded only a slight increase in the AUC compared to Model 1, indicating the existence of a weak interaction of effects in the multiplicative scale. Moreover, it provides a more detailed map of HPV non-vaccination (Table III and Supplemental Tables S1, S2 and S3). The highest risk, 3.5 times higher than that of girls with native parents with high income and education residing in big cities (AR=0.11), was found among girls whose parents had a low income, high education and an immigrant background, living in a rural area (AR=0.39).

**Table II.** Results of the analysis of individual heterogeneity and discriminatory accuracy using AIHDA (Models 1 and 2) and multilevel logistic regression (MAIHDA, Model 3).

		**Model 1 OR (99% CI)**	**Model 2 OR (99% CI)**	**Model 3 OR (99% CI)**
Income	High	Reference		Reference
	Medium	1.24 (1.20–1.28)		1.26 (1.23–1.30)
	Low	1.70 (1.64–1.76)		1.73 (1.69–1.78)
Parental education	High	Reference		Reference
	Low	1.14 (1.11–1.17)		1.13 (1.11–1.15)
Parental country of birth	Native	Reference		Reference
	Mixed	1.45 (1.40–1.50)		1.41 (1.38–1.45)
	Immigrant	1.58 (1.53–1.63)		1.53 (1.50–1.57)
Place of residence	Big cities	Reference		Reference
	Small cities	0.99 (0.97–1.02)		1.04 (0.93–1.17)
	Rural	1.12 (1.07–1.16)		1.13 (0.99–1.26)
AUC		0.605	0.610	0.634
Δ-AUC			0.005	0.024
Variance	County			0.02 (0.01–0.05)
	Municipality			0.12 (0.10–0.14)
ICC	County			0.57%
	Municipality			4.01%

In model 1, HPV non-vaccination was modelled as a function of income, parental education, parental country of birth and place of residence. Model 2 includes the same information in the form of multi-categorical strata (see Table 3 and Supplemental Material). Model 3 expands on Model 1 by adding random effects for the county and municipality levels. Values are ORs with 99% CIs if not otherwise specified.

AIHDA: analysis of individual heterogeneity and discriminatory accuracy; AUC: area under the receiver operating characteristics curve; Δ-AUC: incremental change in the AUC value between the models using model 1 as reference; ICC: intraclass correlation coefficient; OR: odds ratio; CI: confidence interval.

**Table III.** The five strata with the highest and lowest absolute risk (AR) of HPV non-vaccination during 2013–2020, as well as the eight strata with 50% of all the cases (*n*=58,640) among 311,656 girls in 54 multi-categorical strata living in Sweden in 2010.

**Income**	**Parental education**	**Parental country of birth**	**Place of residence**	**Predicted cases**	**Individuals**	**AR (99% CI)**
*The five strata with the highest risk*:						
Low	High	Immigrant	Rural	555	218	0.39 (0.34–0.44)
Low	High	Immigrant	Big city	7755	2548	0.33 (0.31–0.34)
Low	High	Mixed	Small city	1329	409	0.31 (0.28–0.33)
Low	Low	Immigrant	Rural	1715	517	0.30 (0.27–0.32)
Low	High	Mixed	Rural	572	169	0.30 (0.25–0.33)
Overall				11,962	3861	0.32
Share of the total number of cases 7% (3861/58,640)						
*The five strata with the lowest risk*:						
High	High	Native	Rural	2271	316	0.14 (0.12–0.15)
Middle	High	Native	Small city	14,392	1937	0.13 (0.13–0.14)
Middle	High	Native	Big city	25,887	3414	0.13 (0.13–0.13)
High	High	Native	Small city	9081	1091	0.12 (0.11–0.12)
High	High	Native	Big city	39,884	4249	0.11 (0.10–0.11)
Overall				91,515	11,007	0.12
Share of the total number of cases 19% (11,007/58,640)						
*The eight strata with 51% of the cases*:						
Low	Low	Immigrant	Big cities	21,342	5648	0.26 (0.26–0.27)
High	High	Native	Big cities	39,884	4249	0.11 (0.10–0.11)
Low	Low	Native	Big cities	17,219	3993	0.23 (0.22–0.24)
Low	Low	Native	Small cities	16,449	3587	0.22 (0.21–0.22)
Medium	Low	Native	Big cities	20,271	3483	0.17 (0.17–0.18)
Medium	High	Native	Big cities	25,887	3414	0.13 (0.13–0.13)
Medium	Low	Native	Small cities	15,956	2748	0.17 (0.16–0.18)
Low	Low	Immigrant	Big cities	7755	2548	0.33 (0.31–0.34)
Overall				164,763	29,670	0.20
Share of the total number of cases 51% (29,670/58,640)						

Among girls with parents born in Sweden, a social gradient was present, as non-vaccination was less common among those with high parental income and education compared to those with a medium or low income and low education. Among girls with parents born elsewhere, the distribution was more complex. Of the 18 high-education strata, in 13, non-vaccination was more common than in the corresponding (income; place of residence) low-education strata. Of those comprising parental immigration background and high or medium income, three strata showed a higher non-vaccination prevalence than the corresponding medium- or low-income strata. The five strata with the highest prevalence (Table III) comprised girls whose parents had a low income, an immigrant or mixed background, and, in four cases, a high education.

However, the DA of Model 2 was weak (AUC=0.610). In fact, while the non-vaccination prevalence was 2.7 times higher in the five highest-risk strata than in the five lowest-risk strata, the number of cases was 2.9 times higher in the lowest-risk than in the highest-risk strata. The five lowest-risk strata included 19% of all non-vaccination cases, whereas the corresponding number for the five highest-risk strata was 7%. Half of the non-vaccination cases were found in eight strata (Table III).

The geographical analysis (Table II, Model 3, and Tables IV and V), adjusted for the socio-economic variables, shows that the geographical information only adds a very slight increase in the AUC of model 2 (Δ-AUC=0.024). It also shows that 4% of the adjusted differences in the propensity of non-vaccination were located at the municipality level. The differences between counties were without relevance (ICC=0.57%).

**Table IV.** Geographical analysis adjusted for parental income, education, country of birth and place of residence.

	**Individuals**	**Adjusted** ^a^ **predicted cases**	**AR (99% CI)** ^a^
*The five counties with the highest risk*:			
Dalarna	8467	1312	0.16 (0.14–0.17)
Norrbotten	7077	991	0.14 (0.13–0.16)
Stockholm	75,744	10,301	0.14 (0.13–0.14)
Blekinge	4637	626	0.14 (0.12–0.15)
Västerbotten	7935	1047	0.13 (0.12–0.14)
Overall	103,860	14,278	0.14
Share of the total number of cases 38% (14,278/37,505)			
*The five counties with the lowest risk*:			
Västra Götaland	51,388	5499	0.11 (0.10–0.11)
Hallands	10,627	1126	0.11 (0.10–0.11)
Östergötland	13,851	1441	0.10 (0.10–0.11)
Uppsala	11,569	1180	0.10 (0.09–0.11)
Värmland	7768	699	0.09 (0.08–0.10)
Overall	9945	95,203	0.10
Share of the total number of cases 27% (9945/37,505)			
*The three counties with 55% of the cases*:			
Stockholm	75,744	10,301	0.14 (0.13-0.14)
Västra Götaland	51,388	5499	0.11 (0.10-0.11)
Skåne	41,491	4813	0.12 (0.11-0.12)
Overall	168,623	20,613	0.12
Share of the total number of cases 55% (20,613/37,505)			

Information is presented for the five counties with the highest and the five counties with the lowest risk for HPV non-vaccination during 2013–2020, and the three counties showing 55% of all the predicted cases (*n*=37,383), among 311,656 girls in living in Sweden in 2010.

a The figures are obtained from the multilevel analysis (Model 3) and are adjusted for parental education, income, country of birth and place of residence.

**Table V.** Geographical analysis adjusted for parental income, education, country of birth and place of residence.

	**Individuals**	**Adjusted** ^a^ **predicted cases**	**AR (99% CI)** ^a^
*The five municipalities with the highest risk*:			
Storuman	161	41	25.19 (20.69–29.07)
Nybro	531	127	23.96 (17.55–29.74)
Upplands-Bro	1016	241	23.73 (18.51–30.88)
Munkfors	101	24	23.50 (17.00–31.00)
Gagnef	354	81	22.97 (19.39–28.10)
Overall	2163	514	23.87
Share of the total number of cases 1.4% (514/37,383)			
*The five municipalities with the lowest risk*:			
Forshaga	370	23	6.26 (3.18–9.87)
Säffle	414	26	6.18 (5.14–7.21)
Finspång	573	35	6.09 (5.04–7.97)
Emmaboda	228	13	5.83 (4.08–7.06)
Gotland	1648	80	4.88 (3.01–6.73)
Overall	3233	177	5.85
Share of the total number of cases 0.5% (177/37,383)			
*The 37 municipalities with 50% of the cases*:			
Stockholm	27,098	2952	10.89 (8.00–13.42)
Göteborg	15,384	1569	10.20 (8.46–12.57)
Malmö	9133	1304	14.28 (13.06–15.49)
Uppsala	6536	417	6.37 (4.68–8.62)
Linköping	4805	517	10.75 (8.01–12.74)
Västerås	4559	740	16.24 (14.38–17.61)
Örebro	4523	545	12.04 (10.04–14.50)
Jönköping	4352	573	13.17 (9.88–16.66)
Norrköping	4344	296	6.80 (6.12–7.70)
Huddinge	4255	450	10.58 (6.15–14.40)
Helsingborg	4186	479	11.45 (9.54–13.36)
Nacka	4178	482	11.54 (9.15–13.83)
Umeå	3739	440	11.76 (8.97–15.11)
Lund	3631	478	13.16 (11.11–15.29)
Borås	3339	269	8.05 (5.36–12.89)
Kungsbacka	3315	258	7.80 (6.48–9.51)
Eskilstuna	3279	527	16.08 (12.18–21.02)
Sundsvall	3257	288	8.85 (6.56–11.23)
Botkyrka	3239	438	13.53 (10.52–17.06)
Haninge	3004	529	17.61 (12.03–23.97)
Halmstad	3001	227	7.58 (6.37–8.70)
Gävle	2976	353	11.85 (9.30–14.61)
Södertälje	2970	329	11.07 (8.15–14.52)
Sollentuna	2929	335	11.45 (9.49–15.10)
Täby	2877	564	19.62 (14.12–24.79)
Växjö	2818	429	15.22 (12.85–17.72)
Kristianstad	2560	376	14.69 (13.29–16.03)
Järfälla	2553	303	11.86 (8.51–15.86)
Karlstad	2483	246	9.93 (7.46–12.14)
Mölndal	2360	222	9.42 (8.36–10.19)
Luleå	2285	285	12.49 (10.09–14.79)
Skellefteå	2145	239	11.15 (9.06–14 .31)
Karlskrona	2125	413	19 .45 (15.63–22.78)
Varberg	1978	186	9.42 (8.19–11.18)
Östersund	1973	178	9.03 (6.83–11.90)
Kalmar	1939	179	9.21 (6.47–13.78)
Tyresö	1914	287	15.01 (10.13–19.10)
Overall	162,042	18,704	11.79
Share of the total number of cases 50% (18,704/37,383)			

Information is presented for the five municipalities with the highest and the five municipalities with the lowest risk for HPV non-vaccination during 2013–2020, and the 37 municipalities showing 50% of all predicted cases (*n*=37,383), among 311,656 girls in living in Sweden in 2010.

a The figures are obtained from the multilevel analysis (Model 3) and are adjusted for parental education, income, country of birth and place of residence.

The AR of 25.19 in the highest-risk municipality was 5.2 times higher than the AR of 4.88 in the lowest-risk municipality. The five highest-risk municipalities included 1.8% of all the adjusted number of cases in the population, while the corresponding number for the five lowest-risk municipalities was 0.5%. Of all the 290 municipalities, 37 shared 50% of all adjusted cases of HPV non-vaccination.

## Discussion

This nationwide register study shows between-group disparities in the average prevalence of HPV non-vaccination in Sweden. Girls with parents born outside of Sweden and with a lower income or education had a higher probability of non-vaccination than those whose parents had a high income and education and were born in Sweden. This is in alignment with previous research indicating a higher probability of non-vaccination among groups with low income, low education and immigration background [9]. However, our more detailed multi-categorical mapping shows that non-vaccination prevalence was 3.5 times higher among girls whose parents had a low income, high education and immigration background, living in a rural area, compared to the reference stratum. Moreover, among further heterogeneities, we observed that while a social gradient was found among girls with parents born in Sweden, among those with parents both outside of Sweden, several strata with higher income or education showed a higher non-vaccination prevalence than those with lower income or education. This heterogeneous association between income, education and HPV vaccination uptake corresponds to some degree with previous research indicating a higher degree of HPV vaccine hesitancy among highly educated parents in Sweden [7], although high parental education is also associated with higher HPV vaccination uptake [9]. This study confirms the latter, but not in immigrated groups. Meanwhile, and as expected, we observed geographical differences in average risk, most notably at the municipality level.

In a Swedish study of disparities in cervical screening associated with place of birth, these were largely explained by socio-economic rather than cultural or language factors [12]. The results of the present study suggest that such socio-economic aspects likely interact with other issues. A study of attitudes towards HPV vaccination and cervical screening among immigrated women [22] points to barriers including language problems and a lack of knowledge about HPV and about navigation within the Swedish health-care system [22]. Lack of knowledge or information about HPV is not isolated to immigrated populations, however [5,8,23], and it is unclear whether these factors explain the higher non-vaccination prevalence in groups with higher education or income. Other factors associated with HPV non-vaccination in Sweden [5,6] and elsewhere [8] involve trust in vaccinations, health-care providers and the pharmaceutical industry [5,6,8,23]. This issue is actualised in the contemporary context where health-related information is often sought online, where content may contradict information provided by established health-care institutions [24]. Other factors noted to influence decision making include concerns with the vaccine’s safety or efficacy [8] or perceived (in)compatibility with sexual or other ways of life [5,8,23], concern that vaccination may be conducive to risky behaviour through creating a false sense of security [8], and practices or attitudes of health-care professionals [6]. In addition, it may be worth noting that foregone health care among immigrant groups has been associated with experiences of discrimination [25], which may impact levels of trust [26]. Furthermore, it should also be observed that while the risk of cervical cancer is lower in some immigrant groups [13], this should not impact the universal vaccination coverage goal.

Meanwhile, a central contribution of the AIHDA approach is the assessments of the DA of the categorisations used, without which measures of average risk may convey a risk of stigmatisation of ‘high-risk’ groups, of creating false expectations in ‘low-risk’ groups and of ineffective interventions due to over- or undertreatment [21]. Our analysis shows that the DA of the geographical and socio-economic information was weak. In fact, and paradoxically [27], many cases of non-vaccination occurred in groups with low average risk.

This low DA co-exists with a universal vaccination programme which has alleviated previously greater disparities by largely benefitting less privileged groups in Sweden [9]. In that context, our results suggest that interventions to improve HPV vaccination should be directed to the whole population, while targeted or tailored intervention considering circumstances or characteristics of groups may simultaneously be warranted. Thus, we underline the importance of providing adequate information about HPV vaccination in Swedish and other languages [22,23], potentially in combination with other locally designed interventions [28], and of health-care professionals displaying sensitivity to patients’ or parents’ questions or needs [22,23] while avoiding forms of interaction which may discourage trust and confidence [26]. Any targeted interventions should be evaluated with both specificity and sensitivity in mind. While the existence of false-positives may be a lesser problem than that of false-negatives, the former can actualise the issue of stigmatisation.

It should furthermore be noted that an increased uptake of HPV vaccination among boys has been predicted to improve the resilience of HPV infection prevention overall [29].

### Limitations

Being observational, this study does not enable the drawing of conclusions about causal relationships. In addition, some multi-categorical strata were rather small, which is reflected in the wide CIs conveying a limited reliability of some point estimates. Furthermore, the categorisation based on country of birth can be seen as simplistic and insufficient, as it disregards large heterogeneities within the group [30] by conflating, for example, immigrants from Nordic countries with refugees from other continents. Disaggregation into more distinct areas of origin proved difficult, however, as this would considerably reduce the strata size. Furthermore, while the HPV vaccination programme today includes boys [29], it did not do so during the study period, which is why our study only includes girls.

## Conclusions

This study corroborates previous findings by indicating a higher prevalence of HPV non-vaccination among girls with immigrated parents with low income and low education who live in rural areas, while also showing geographical differences between municipalities. We provide, however, a more precise mapping of socio-economic disparities in HPV non-vaccination in Sweden. We also observed that the DA of the categorisations used was low, and that many cases of non-vaccination are found in low-risk groups. These findings suggest that interventions aiming to increase HPV vaccination should be directed towards the whole population, while simultaneously considering characteristics of particular groups. We underline the importance of providing adequate information about HPV vaccination in Swedish and other languages, and of health-care professionals displaying sensitivity to patients’ questions or needs while avoiding forms of communication which may discourage trust and confidence.

## Supplemental Material

sj-docx-1-sjp-10.1177_14034948221075410 – Supplemental material for Mapping sociodemographic and geographical differences in human papillomavirus non-vaccination among young girls in Sweden Click here for additional data file. Supplemental material, sj-docx-1-sjp-10.1177_14034948221075410 for Mapping sociodemographic and geographical differences in human papillomavirus non-vaccination among young girls in Sweden by Maria Wemrell, Raquel Perez Vicente and Juan Merlo in Scandinavian Journal of Public Health

sj-docx-2-sjp-10.1177_14034948221075410 – Supplemental material for Mapping sociodemographic and geographical differences in human papillomavirus non-vaccination among young girls in Sweden Click here for additional data file. Supplemental material, sj-docx-2-sjp-10.1177_14034948221075410 for Mapping sociodemographic and geographical differences in human papillomavirus non-vaccination among young girls in Sweden by Maria Wemrell, Raquel Perez Vicente and Juan Merlo in Scandinavian Journal of Public Health
