# Supplementary figures and images for: Mapping sociodemographic and geographical differences in human papillomavirus non-vaccination among young girls in Sweden
Source: Scand J Public Health. 2022 Feb 4;51(2):288–95. doi: 10.1177/14034948221075410 (PMC9969304; doi:10.1177/14034948221075410)

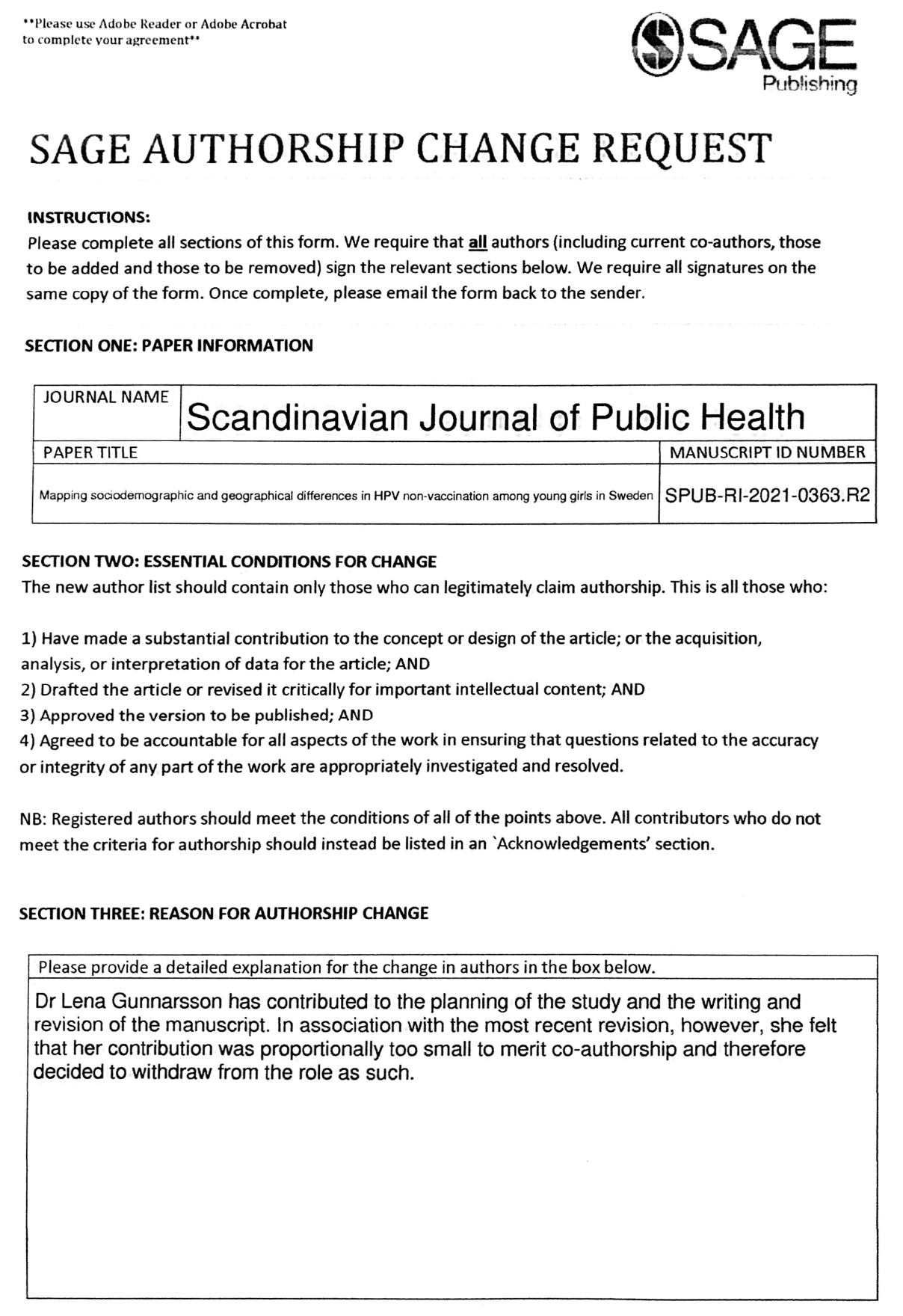


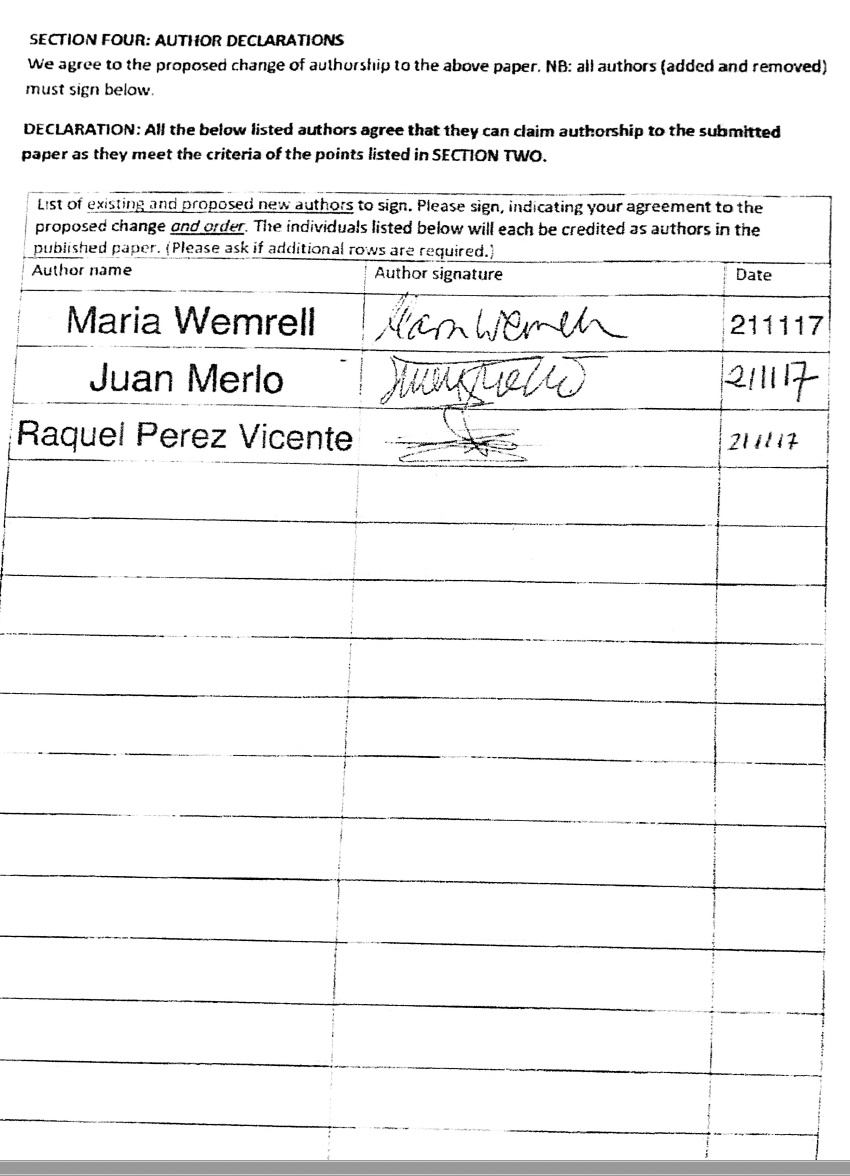


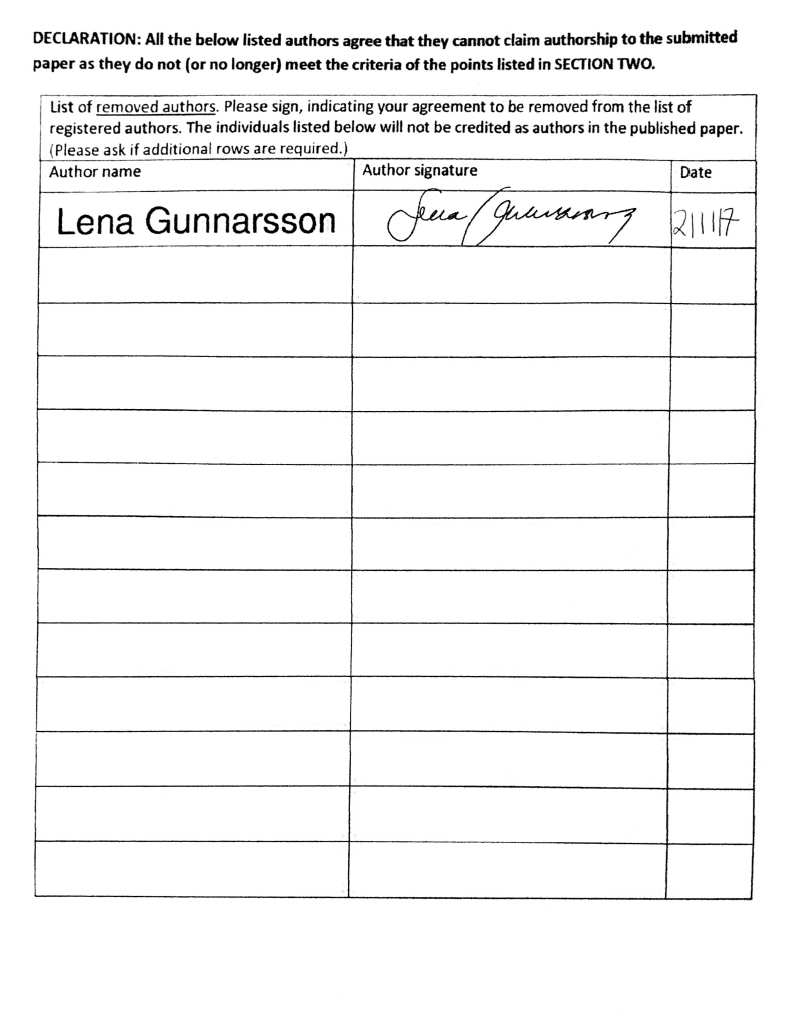

Supplement: sj-docx-1-sjp-10.1177_14034948221075410 – Supplemental material for Mapping sociodemographic and geographical differences in human papillomavirus non-vaccination among young girls in Sweden [file sj-docx-1-sjp-10.1177_14034948221075410.docx]
