# Supplementary material for: Mapping sociodemographic and geographical differences in human papillomavirus non-vaccination among young girls in Sweden
Source: Scand J Public Health. 2022 Feb 4;51(2):288–95. doi: 10.1177/14034948221075410 (PMC9969304; doi:10.1177/14034948221075410)
Supplement: sj-docx-2-sjp-10.1177_14034948221075410 – Supplemental material for Mapping sociodemographic and geographical differences in human papillomavirus non-vaccination among young girls in Sweden [file sj-docx-2-sjp-10.1177_14034948221075410.docx]

**Supplementary material**


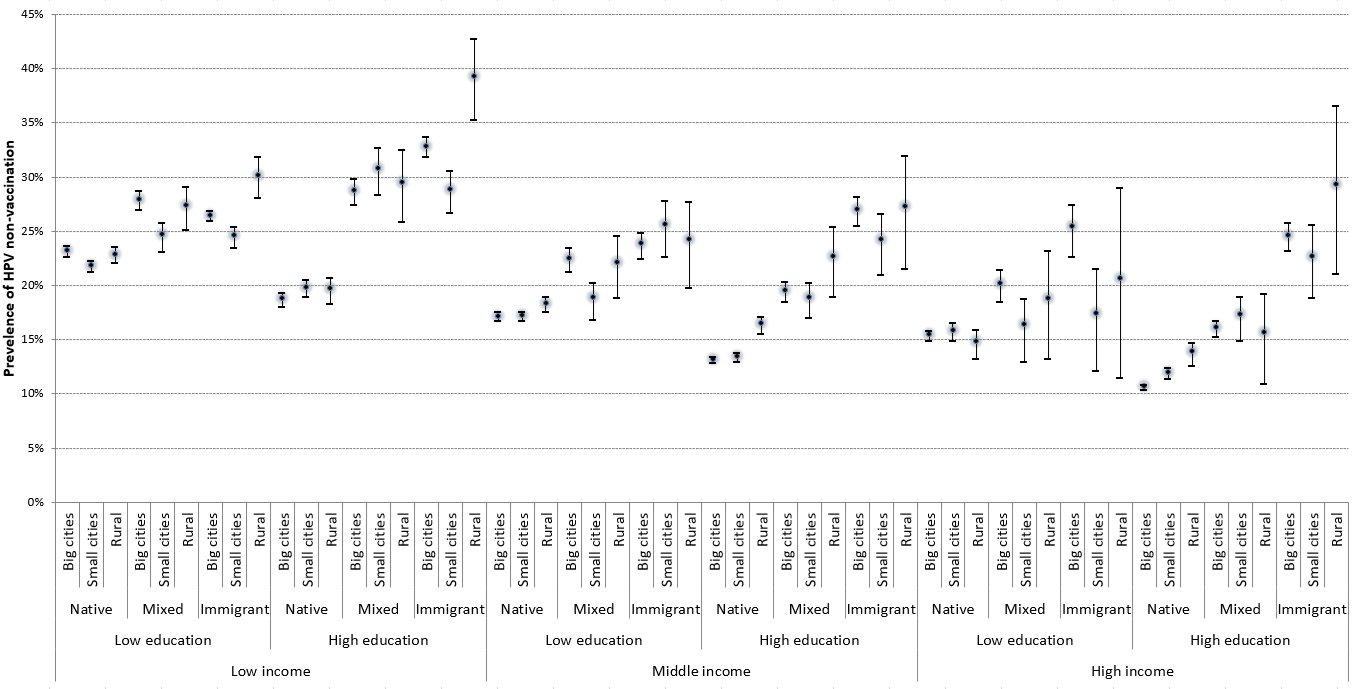
**S1.** Prevalence of HPV non-vaccination across 54 strata defined by demographic and socioeconomic variables, among 311,656 girls living in Sweden in 2010.

**S2.** Results of the multicategorical AIHDA showing the absolute risk (AR) (i.e., prevalence) of HPV non-vaccination with 99% confidence intervals (CIs) across all multicategorical strata. The odds ratios (ORs) using the stratum #46 as reference are also indicated. The strata are categorized by quintiles of risk into green (AR=0–0.16), light green (AR=0.17–2.00), yellow (AR=2.10–2.30), orange (AR=0.24–0.27) and red colour (AR=0.28–0.39).

| **Stratum ID #** |  |  |  |  | OR (99% CI) | AR (99% CI) | Cases (n) | Total (n) |
| --- | --- | --- | --- | --- | --- | --- | --- | --- |
| 1 |  | Low edu | Native | Big cities | 2.53 (2.38–2.70) | 0.23 (0.23–0.24) | 3993 | 17,219 |
| 2 | Low |  |  | Small cities | 2.34 (2.19–2.49) | 0.22 (0.21–0.22) | 3587 | 16,449 |
| 3 | income |  |  | Rural areas | 2.49 (2.30–2.69) | 0.23 (0.22–0.23) | 1989 | 8685 |
| 4 |  |  | Mixed | Big cities | 3.26 (3.01–3.53) | 0.28 (0.27–0.29) | 1976 | 7066 |
| 5 |  |  |  | Small cities | 2.75 (2.43–3.10) | 0.25 (0.23–0.26) | 687 | 2785 |
| 6 |  |  |  | Rural areas | 3.16 (2.70–3.70) | 0.27 (0.25–0.29) | 398 | 1453 |
| 7 |  |  | Imm. | Big cities | 3.02 (2.85–3.20) | 0.26 (0.26–0.27) | 5648 | 21,342 |
| 8 |  |  |  | Small cities | 2.73 (2.49–3.00) | 0.25 (0.23–0.25) | 1257 | 5112 |
| 9 |  |  |  | Rural areas | 3.62 (3.14–4.17) | 0.30 (0.28–0.32) | 517 | 1715 |
| 10 |  | High edu | Native | Big cities | 1.94 (1.79–2.10) | 0.19 (0.18–0.19) | 1855 | 9876 |
| 11 |  |  |  | Small cities | 2.08 (1.90–2.27) | 0.20 (0.19–0.20) | 1268 | 6391 |
| 12 |  |  |  | Rural areas | 2.06 (1.81–2.34) | 0.20 (0.18–0.21) | 556 | 2819 |
| 13 |  |  | Mixed | Big cities | 3.39 (3.07–3.74) | 0.29 (0.27–0.30) | 1176 | 4085 |
| 14 |  |  |  | Small cities | 3.73 (3.18–4.37) | 0.31 (0.28–0.33) | 409 | 1329 |
| 15 |  |  |  | Rural areas | 3.52 (2.77–4.47) | 0.30 (0.26–0.32) | 169 | 572 |
| 16 |  |  | Imm. | Big cities | 4.10 (3.81–4.42) | 0.33 (0.32–0.34) | 2548 | 7755 |
| 17 |  |  |  | Small cities | 3.40 (2.93–3.95) | 0.29 (0.27–0.31) | 451 | 1562 |
| 18 |  |  |  | Rural areas | 5.43 (4.32–6.81) | 0.39 (0.35–0.43) | 218 | 555 |
| 19 |  | Low edu | Native | Big cities | 1.74 (1.63–1.85) | 0.17 (0.17–0.17) | 3483 | 20,271 |
| 20 | Medium |  |  | Small cities | 1.74 (1.63–1.87) | 0.17 (0.17–0.18) | 2748 | 15,956 |
| 21 | income |  |  | Rural areas | 1.89 (1.73–2.06) | 0.18 (0.17–0.19) | 1327 | 7217 |
| 22 |  |  | Mixed | Big cities | 2.44 (2.19–2.72) | 0.23 (0.21–0.23) | 874 | 3878 |
| 23 |  |  |  | Small cities | 1.95 (1.62–2.34) | 0.19 (0.17–0.20) | 258 | 1368 |
| 24 |  |  |  | Rural areas | 2.39 (1.84–3.10) | 0.22 (0.19–0.24) | 128 | 578 |
| 25 |  |  | Imm. | Big cities | 2.63 (2.35–2.94) | 0.24 (0.22–0.25) | 805 | 3373 |
| 26 |  |  |  | Small cities | 2.88 (2.33–3.57) | 0.26 (0.23–0.28) | 206 | 805 |
| 27 |  |  |  | Rural areas | 2.68 (1.92–3.75) | 0.24 (0.20–0.28) | 79 | 326 |
| 28 |  | High edu | Native | Big cities | 1.27 (1.20–1.36) | 0.13 (0.13–0.13) | 3414 | 25,887 |
| 29 |  |  |  | Small cities | 1.30 (1.21–1.41) | 0.13 (0.13–0.14) | 1937 | 14,392 |
| 30 |  |  |  | Rural areas | 1.66 (1.49–1.84) | 0.16 (0.15–0.17) | 828 | 5021 |
| 31 |  |  | Mixed | Big cities | 2.04 (1.84–2.26) | 0.20 (0.18–0.20) | 942 | 4818 |
| 32 |  |  |  | Small cities | 1.96 (1.64–2.35) | 0.19 (0.17–0.20) | 267 | 1410 |
| 33 |  |  |  | Rural areas | 2.46 (1.84–3.28) | 0.23 (0.19–0.25) | 105 | 463 |
| 34 |  |  | Imm. | Big cities | 3.10 (2.77–3.47) | 0.27 (0.25–0.28) | 829 | 3070 |
| 35 |  |  |  | Small cities | 2.68 (2.10–3.41) | 0.24 (0.21–0.27) | 154 | 636 |
| 36 |  |  |  | Rural areas | 3.15 (2.12–4.66) | 0.27 (0.22–0.32) | 60 | 220 |
| 37 |  | Low edu | Native | Big cities | 1.53 (1.42–1.65) | 0.15 (0.15–0.16) | 2031 | 13,133 |
| 38 | High |  |  | Small cities | 1.58 (1.42–1.77) | 0.16 (0.15–0.16) | 759 | 4778 |
| 39 | income |  |  | Rural areas | 1.47 (1.22–1.76) | 0.15 (0.13–0.16) | 252 | 1693 |
| 40 |  |  | Mixed | Big cities | 2.12 (1.82–2.47) | 0.20 (0.18–0.21) | 384 | 1901 |
| 41 |  |  |  | Small cities | 1.64 (1.15–2.33) | 0.16 (0.13–0.19) | 65 | 397 |
| 42 |  |  |  | Rural areas | 1.94 (1.16–3.25) | 0.19 (0.13–0.23) | 31 | 165 |
| 43 |  |  | Imm. | Big cities | 2.86 (2.34–3.49) | 0.25 (0.23–0.27) | 235 | 925 |
| 44 |  |  |  | Small cities | 1.77 (1.05–2.98) | 0.17 (0.12–0.21) | 30 | 172 |
| 45 |  |  |  | Rural areas | 2.18 (0.98–4.87) | 0.21 (0.11–0.29) | 13 | 63 |
| 46 |  | High edu | Native | Big cities | Reference | 0.11 (0.10–0.11) | 4249 | 39,884 |
| 47 |  |  |  | Small cities | 1.15 (1.04–1.26) | 0.12 (0.11–0.12) | 1091 | 9081 |
| 48 |  |  |  | Rural areas | 1.36 (1.15–1.59) | 0.14 (0.13–0.15) | 316 | 2271 |
| 49 |  |  | Mixed | Big cities | 1.61 (1.46–1.78) | 0.16 (0.15–0.17) | 997 | 6179 |
| 50 |  |  |  | Small cities | 1.75 (1.38–2.23) | 0.17 (0.15–0.19) | 145 | 838 |
| 51 |  |  |  | Rural areas | 1.56 (0.93–2.61) | 0.16 (0.11–0.19) | 30 | 191 |
| 52 |  |  | Imm. | Big cities | 2.74 (2.44–3.08) | 0.25 (0.23–0.26) | 737 | 2990 |
| 53 |  |  |  | Small cities | 2.47 (1.83–3.33) | 0.23 (0.19–0.26) | 97 | 427 |
| 54 |  |  |  | Rural areas | 3.49 (2.02–6.00) | 0.29 (0.21–0.37) | 32 | 109 |

**S3.** The multicategorical strata presented according to the number of HPV non-vaccination cases, showing the cumulative numbers of cases as well as the absolute risk (AR) and odds ratios (ORs) with 99% confidence intervals (CIs), of non-vaccination across the 54 strata.

| **Stratum**  **ID #** | **Income** | **Education** | **COB** | **Place** | **OR (99% CI)** | **AR (99% CI)** | **Total**  **(n)** | **Cases (n)** | **Cum.**  **(%)** |
| --- | --- | --- | --- | --- | --- | --- | --- | --- | --- |
| 7 | Low | Low | Immigrant | Big cities | 3.02 (2.85–3.20) | 0.26 (0.26–0.27) | 21,342 | 5648 | 9.6 |
| 46 | High | High | Native | Big cities | Reference | 0.11 (0.10–0.11) | 39,884 | 4249 | 16.9 |
| 1 | Low | Low | Native | Big cities | 2.53 (2.38–2.70) | 0.23 (0.23–0.24) | 17,219 | 3993 | 23.7 |
| 2 | Low | Low | Native | Small cities | 2.34 (2.19–2.49) | 0.22 (0.21–0.22) | 16,449 | 3587 | 29,8 |
| 19 | Medium | Low | Native | Big cities | 1.74 (1.63–1.85) | 0.17 (0.17–0.17) | 20,271 | 3483 | 35.7 |
| 28 | Medium | High | Native | Big cities | 1.27 (1.20–1.36) | 0.13 (0.13–0.13) | 25,887 | 3414 | 41.6 |
| 20 | Medium | Low | Native | Small cities | 1.74 (1.63–1.87) | 0.17 (0.17–0.18) | 15,956 | 2748 | 46.3 |
| 16 | Low | Low | Immigrant | Big cities | 4.10 (3.81–4.42) | 0.33 (0.32–0.34) | 7755 | 2548 | 50.6 |
| 37 | High | Low | Native | Big cities | 1.53 (1.42–1.65) | 0.15 (0.15–0.16) | 13,133 | 2031 | 54.1 |
| 3 | Low | Low | Native | Rural areas | 2.49 (2.30–2.69) | 0.23 (0.22–0.23) | 8685 | 1989 | 57.5 |
| 4 | Low | Low | Mixed | Big cities | 3.26 (3.01–3.53) | 0.28 (0.27–0.29) | 7066 | 1976 | 60.8 |
| 29 | Medium | High | Native | Small cities | 1.30 (1.21–1.41) | 0.13 (0.13–0.14) | 14,392 | 1937 | 64.1 |
| 10 | Low | High | Native | Big cities | 1.94 (1.79–2.10) | 0.19 (0.18–0.19) | 9876 | 1855 | 67.3 |
| 21 | Medium | Low | Native | Rural areas | 1.89 (1.73–2.06) | 0.18 (0.17–0.19) | 7217 | 1327 | 69.6 |
| 11 | Low | High | Native | Small cities | 2.08 (1.90–2.27) | 0.2 (0.19–0.20) | 6391 | 1268 | 71.7 |
| 8 | Low | Low | Immigrant | Small cities | 2.73 (2.49–3.00) | 0.25 (0.23–0.25) | 5112 | 1257 | 73.9 |
| 13 | Low | High | Mixed | Big cities | 3.39 (3.07–3.74) | 0.29 (0.27–0.30) | 4085 | 1176 | 75.9 |
| 47 | High | High | Native | Small cities | 1.15 (1.04–1.26) | 0.12 (0.11–0.12) | 9081 | 1091 | 77.7 |
| 49 | High | High | Mixed | Big cities | 1.61 (1.46–1.78) | 0.16 (0.15–0.17) | 6179 | 997 | 79.4 |
| 31 | Medium | High | Mixed | Big cities | 2.04 (1.84–2.26) | 0.20 (0.18–0.20) | 4818 | 942 | 81.0 |
| 22 | Medium | Low | Mixed | Big cities | 2.44 (2.19–2.72) | 0.23 (0.21–0.23) | 3878 | 874 | 82.5 |
| 34 | Medium | High | Immigrant | Big cities | 3.1 (2.77–3.47) | 0.27 (0.25–0.28) | 3070 | 829 | 83.9 |
| 30 | Medium | High | Native | Rural areas | 1.66 (1.49–1.84) | 0.16 (0.15–0.17) | 5021 | 828 | 85.3 |
| 25 | Medium | Low | Immigrant | Big cities | 2.63 (2.35–2.94) | 0.24 (0.22–0.25) | 3373 | 805 | 86.7 |
| 38 | High | Low | Native | Small cities | 1.58 (1.42–1.77) | 0.16 (0.15–0.16) | 4778 | 759 | 88.0 |
| 52 | High | High | Immigrant | Big cities | 2.74 (2.44–3.08) | 0.25 (0.23–0.26) | 2990 | 737 | 89.3 |
| 5 | Low | Low | Mixed | Small cities | 2.75 (2.43–3.10) | 0.25 (0.23–0.26) | 2785 | 687 | 90.4 |
| 12 | Low | High | Native | Rural areas | 2.06 (1.81–2.34) | 0.2 (0.18–0.21) | 2819 | 556 | 91.4 |
| 9 | Low | Low | Immigrant | Rural areas | 3.62 (3.14–4.17) | 0.3 (0.28–0.32) | 1715 | 517 | 92.3 |
| 17 | Low | High | Immigrant | Small cities | 3.4 (2.93–3.95) | 0.29 (0.27–0.31) | 1562 | 451 | 93.0 |
| 14 | Low | High | Mixed | Small cities | 3.73 (3.18–4.37) | 0.31 (0.28–0.33) | 1329 | 409 | 93.7 |
| 6 | Low | Low | Mixed | Rural areas | 3.16 (2.70–3.70) | 0.27 (0.25–0.29) | 1453 | 398 | 94.4 |
| 40 | High | Low | Mixed | Big cities | 2.12 (1.82–2.47) | 0.2 (0.18–0.21) | 1901 | 384 | 95.1 |
| 48 | High | High | Native | Rural areas | 1.36 (1.15–1.59) | 0.14 (0.13–0.15) | 2271 | 316 | 95.6 |
| 32 | Medium | High | Mixed | Small cities | 1.96 (1.64–2.35) | 0.19 (0.17–0.20) | 1410 | 267 | 96.1 |
| 23 | Medium | Low | Mixed | Small cities | 1.95 (1.62–2.34) | 0.19 (0.17–0.20) | 1368 | 258 | 96.5 |
| 39 | High | Low | Native | Rural areas | 1.47 (1.22–1.76) | 0.15 (0.13–0.16) | 1693 | 252 | 96.9 |
| 43 | High | Low | Immigrant | Big cities | 2.86 (2.34–3.49) | 0.25 (0.23–0.27) | 925 | 235 | 97.3 |
| 18 | Low | High | Immigrant | Rural areas | 5.43 (4.32–6.81) | 0.39 (0.35–0.43) | 555 | 218 | 97.7 |
| 26 | Medium | Low | Immigrant | Small cities | 2.88 (2.33–3.57) | 0.26 (0.23–0.28) | 805 | 206 | 98.1 |
| 15 | Low | High | Mixed | Rural areas | 3.52 (2.77–4.47) | 0.3 (0.26–0.32) | 572 | 169 | 98.3 |
| 35 | Medium | High | Immigrant | Small cities | 2.68 (2.10–3.41) | 0.24 (0.21–0.27) | 636 | 154 | 98.6 |
| 50 | High | High | Mixed | Small cities | 1.75 (1.38–2.23) | 0.17 (0.15–0.19) | 838 | 145 | 98.9 |
| 24 | Medium | Low | Mixed | Rural areas | 2.39 (1.84–3.10) | 0.22 (0.19–0.24) | 578 | 128 | 99.1 |
| 33 | Medium | High | Mixed | Rural areas | 2.46 (1.84–3.28) | 0.23 (0.19–0.25) | 463 | 105 | 99.3 |
| 53 | High | High | Immigrant | Small cities | 2.47 (1.83–3.33) | 0.23 (0.19–0.26) | 427 | 97 | 99.4 |
| 27 | Medium | Low | Immigrant | Rural areas | 2.68 (1.92–3.75) | 0.24 (0.20–0.28) | 326 | 79 | 99.6 |
| 41 | High | Low | Mixed | Small cities | 1.64 (1.15–2.33) | 0.16 (0.13–0.19) | 397 | 65 | 99.7 |
| 36 | Medium | High | Immigrant | Rural areas | 3.15 (2.12–4.66) | 0.27 (0.22–0.32) | 220 | 60 | 99.8 |
| 54 | High | High | Immigrant | Rural areas | 3.49 (2.02–6.00) | 0.29 (0.21–0.37) | 109 | 32 | 99.8 |
| 42 | High | Low | Mixed | Rural areas | 1.94 (1.16–3.25) | 0.19 (0.13–0.23) | 165 | 31 | 99.9 |
| 44 | High | Low | Immigrant | Small cities | 1.77 (1.05–2.98) | 0.17 (0.12–0.21) | 172 | 30 | 99.9 |
| 51 | High | High | Mixed | Rural areas | 1.56 (0.93–2.61) | 0.16 (0.11–0.19) | 191 | 30 | 100.0 |
| 45 | High | Low | Immigrant | Rural areas | 2.18 (0.98–4.87) | 0.21 (0.11–0.29) | 63 | 13 | 100.0 |

**S4.** Results of the geographical analysis, showing the absolute risk (AR) (i.e., prevalence) of


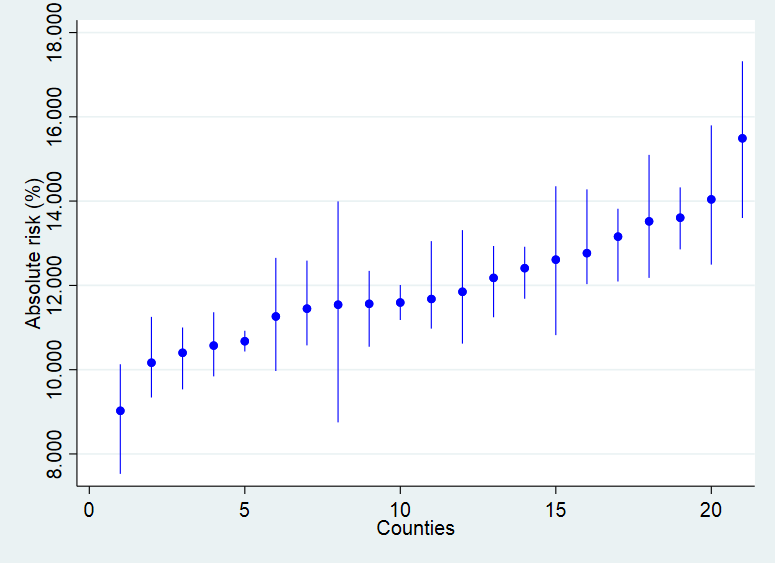
HPV non-vaccination with 99% confidence intervals (CIs) across the 21 counties of Sweden.

**S5.** Results of the geographical analysis, showing the absolute risk (AR) (i.e., prevalence) of

HPV non-vaccination with 99% confidence intervals (CIs), as well as the total number of individuals and the predicted number of cases across the 21 counties in Sweden.

| County | Total  (n) | Predicted cases  (n) | AR (99% CI) |
| --- | --- | --- | --- |
| Blekinge | 4637 | 626 | 0.14 (0.12–0.15) |
| Dalarna | 8467 | 1312 | 0.16 (0.14–0.17) |
| Gävleborg | 8262 | 942 | 0.11 (0.11–0.13) |
| Gotland | 1648 | 190 | 0.12 (0.09–0.14) |
| Halland | 10,627 | 1126 | 0.11 (0.10–0.11) |
| Jämtland | 4072 | 513 | 0.13 (0.11–0.14) |
| Jönköping | 11,139 | 1359 | 0.12 (0.11–0.13) |
| Kalmar | 6632 | 769 | 0.12 (0.11–0.12) |
| Kronoberg | 5931 | 759 | 0.13 (0.12–0.14) |
| Norrbotten | 7077 | 991 | 0.14 (0.13–0.16) |
| Örebro | 8789 | 993 | 0.11 (0.10–0.13) |
| Östergötland | 13,851 | 1441 | 0.10 (0.10–0.11) |
| Skåne | 41,491 | 4813 | 0.12 (0.11–0.12) |
| Södermanland | 9036 | 1057 | 0.12 (0.11–0.13) |
| Stockholm | 75,744 | 10301 | 0.14 (0.13–0.14) |
| Uppsala | 11,569 | 1180 | 0.10 (0.09–0.11) |
| Värmland | 7768 | 699 | 0.09 (0.08–0.10) |
| Västerbotten | 7935 | 1047 | 0.13 (0.12–0.14) |
| Västernorrland | 7675 | 906 | 0.12 (0.11–0.13) |
| Västmanland | 7918 | 982 | 0.12 (0.12–0.13) |
| Västra Götaland | 51,388 | 5499 | 0.11 (0.10–0.11) |

S6. Results of the geographical analysis, showing the absolute risk (AR) (i.e., prevalence) of


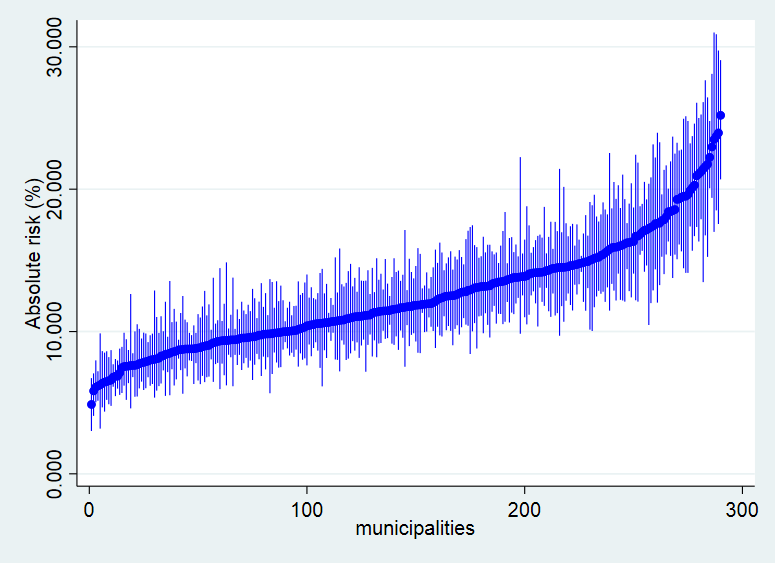
HPV non-vaccination with 99% confidence intervals (CIs), across the 290 municipalities in Sweden.

**S7.** Results of the geographical analysis, showing the absolute risk (AR) (i.e., prevalence) of

HPV non-vaccination with 99% confidence intervals (CIs), as well as the total number of individuals and the predicted number of cases across the 290 municipalities in Sweden.

| Municipality | Total  (n) | Predicted cases (n) | AR (99% CI) |
| --- | --- | --- | --- |
| Ale | 1122 | 150 | 13.40 (11.80–15.42) |
| Alingsås | 1384 | 132 | 9.55 (7.30–12.09) |
| Älmhult | 512 | 71 | 13.80 (11.22–15.58) |
| Älvdalen | 238 | 36 | 15.19 (12.46–18.08) |
| Alvesta | 629 | 91 | 14.53 (12.17–17.61) |
| Älvkarleby | 280 | 28 | 9.83 (8.19–11.15) |
| Älvsbyn | 223 | 25 | 11.11 (9.43–13.56) |
| Åmål | 350 | 50 | 14.20 (11.81–18.75) |
| Aneby | 176 | 15 | 8.79 (6.32–10.43) |
| Ånge | 248 | 42 | 16.99 (15.67–18.97) |
| Ängelholm | 1337 | 176 | 13.18 (11.29–15.56) |
| Arboga | 345 | 35 | 10.06 (8.22–11.79) |
| Åre | 338 | 40 | 11.87 (8.48–15.45) |
| Årjäng | 278 | 36 | 12.92 (8.42–17.34) |
| Arjeplog | 73 | 7 | 9.37 (6.21–14.86) |
| Arvidsjaur | 180 | 26 | 14.39 (11.03–17.72) |
| Arvika | 733 | 47 | 6.46 (4.38–8.52) |
| Åsele | 67 | 7 | 10.81 (9.08–13.16) |
| Askersund | 342 | 38 | 11.04 (8.66–13.77) |
| Åstorp | 582 | 62 | 10.64 (9.94–11.31) |
| Åtvidaberg | 382 | 50 | 13.21 (9.58–16.09) |
| Avesta | 589 | 98 | 16.7 (12.1–21.86) |
| Båstad | 402 | 58 | 14.44 (12.67–15.81) |
| Bengtsfors | 225 | 21 | 9.45 (7.65–11.55) |
| Berg | 228 | 30 | 13.09 (8.81–15.93) |
| Bjurholm | 62 | 7 | 11.57 (9.06–15.15) |
| Bjuv | 537 | 74 | 13.8 (11.97–16.11) |
| Boden | 776 | 86 | 11.05 (9.23–12.79) |
| Bollebygd | 353 | 65 | 18.42 (16.32–20.04) |
| Bollnäs | 783 | 52 | 6.59 (4.78–8.7) |
| Borås | 3339 | 269 | 8.05 (5.36–12.89) |
| Borgholm | 252 | 20 | 8.13 (6.16–9.91) |
| Borlänge | 1601 | 241 | 15.07 (10.04–18.87) |
| Botkyrka | 3239 | 438 | 13.53 (10.52–17.06) |
| Boxholm | 144 | 20 | 14.15 (10.35–16.72) |
| Bräcke | 174 | 21 | 11.95 (9.99–14.15) |
| Bromölla | 395 | 57 | 14.51 (11.78–16.97) |
| Burlöv | 621 | 120 | 19.28 (15.08–23.26) |
| Dals–Ed | 136 | 13 | 9.38 (8.37–10.21) |
| Danderyd | 1559 | 173 | 11.07 (9.02–13.58) |
| Degerfors | 253 | 24 | 9.56 (8.15–11.22) |
| Dorotea | 62 | 7 | 11.71 (7.53–17.14) |
| Eda | 211 | 16 | 7.57 (5.20–9.46) |
| Ekerö | 1251 | 209 | 16.7 (12.19–22.42) |
| Eksjö | 504 | 50 | 9.89 (8.52–11.67) |
| Emmaboda | 228 | 13 | 5.83 (4.08–7.06) |
| Enköping | 1398 | 123 | 8.82 (7.78–9.91) |
| Eskilstuna | 3279 | 527 | 16.08 (12.18–21.02) |
| Eslöv | 1095 | 131 | 11.95 (11.00–13.02) |
| Essunga | 175 | 18 | 10.03 (8.31–12.09) |
| Fagersta | 339 | 65 | 19.32 (16.11–22.71) |
| Falkenberg | 1295 | 99 | 7.63 (6.8–8.47) |
| Falköping | 1010 | 95 | 9.36 (7.75–10.74) |
| Falun | 1883 | 276 | 14.64 (11.86–17.4) |
| Färgelanda | 176 | 17 | 9.69 (8.43–10.94) |
| Filipstad | 262 | 22 | 8.48 (5.52–13.55) |
| Finspång | 573 | 35 | 6.09 (5.04–7.97) |
| Flen | 423 | 43 | 10.16 (7.53–13.52) |
| Forshaga | 370 | 23 | 6.26 (3.18–9.87) |
| Gagnef | 354 | 81 | 22.97 (19.39–28.1) |
| Gällivare | 441 | 96 | 21.75 (15.24–26.44) |
| Gävle | 2976 | 353 | 11.85 (9.3–14.61) |
| Gislaved | 946 | 117 | 12.37 (9.65–15.55) |
| Gnesta | 376 | 39 | 10.42 (9.09–12.38) |
| Gnosjö | 297 | 29 | 9.62 (7.95–11.43) |
| Göteborg | 15,384 | 1569 | 10.20 (8.46–12.57) |
| Götene | 397 | 45 | 11.37 (8.19–15.15) |
| Gotland | 1648 | 80 | 4.88 (3.01–6.73) |
| Grästorp | 201 | 19 | 9.25 (7.72–10.55) |
| Grums | 271 | 29 | 10.57 (7.44–14.11) |
| Gullspång | 130 | 18 | 13.74 (11.33–16.64) |
| Habo | 504 | 80 | 15.92 (12.87–20.5) |
| Håbo | 877 | 60 | 6.87 (5.99–7.97) |
| Hagfors | 240 | 18 | 7.47 (5.68–8.92) |
| Hällefors | 166 | 17 | 10.48 (8.26–13.11) |
| Hallsberg | 451 | 44 | 9.83 (7.34–12.7) |
| Hallstahammar | 471 | 67 | 14.3 (11.42–16.65) |
| Halmstad | 3001 | 227 | 7.58 (6.37–8.70) |
| Hammarö | 606 | 63 | 10.41 (7.62–14) |
| Haninge | 3004 | 529 | 17.61 (12.03–23.97) |
| Haparanda | 296 | 32 | 10.72 (8.03–15.21) |
| Härjedalen | 273 | 24 | 8.72 (7.29–11.30) |
| Härnösand | 753 | 102 | 13.48 (11.25–14.81) |
| Härryda | 1596 | 140 | 8.78 (7.93–9.75) |
| Hässleholm | 1539 | 342 | 22.24 (20.06–24.79) |
| Heby | 360 | 31 | 8.55 (7.35–10.66) |
| Hedemora | 474 | 77 | 16.31 (12.40–20.41) |
| Helsingborg | 4186 | 479 | 11.45 (9.54–13.36) |
| Herrljunga | 306 | 36 | 11.68 (9.57–13.99) |
| Hjo | 250 | 19 | 7.75 (6.00–10.19) |
| Hofors | 254 | 20 | 7.83 (5.89–9.92) |
| Höganäs | 910 | 134 | 14.78 (13.32–16.46) |
| Högsby | 136 | 19 | 14.13 (11.98–16.42) |
| Höör | 563 | 90 | 16.03 (12.22–18.67) |
| Hörby | 485 | 34 | 7.09 (5.55–8.80) |
| Huddinge | 4255 | 450 | 10.58 (6.15–14.40) |
| Hudiksvall | 1172 | 159 | 13.56 (10.68–18.39) |
| Hultsfred | 281 | 25 | 8.94 (6.88–11.76) |
| Hylte | 321 | 50 | 15.66 (13.10–18.23) |
| Järfälla | 2553 | 303 | 11.86 (8.51–15.86) |
| Jokkmokk | 111 | 18 | 15.78 (11.47–22.54) |
| Jönköping | 4352 | 573 | 13.17 (9.88–16.66) |
| Kalix | 444 | 48 | 10.78 (7.20–15.83) |
| Kalmar | 1939 | 179 | 9.21 (6.47–13.78) |
| Karlsborg | 193 | 16 | 8.40 (5.48–12.09) |
| Karlshamn | 892 | 112 | 12.55 (10.19–15.26) |
| Karlskoga | 855 | 109 | 12.70 (9.78–15.72) |
| Karlskrona | 2125 | 413 | 19.45 (15.63–22.78) |
| Karlstad | 2483 | 246 | 9.93 (7.46–12.14) |
| Katrineholm | 955 | 120 | 12.62 (10.44–15.06) |
| Kävlinge | 1297 | 150 | 11.59 (9.66–14.18) |
| Kil | 419 | 67 | 15.97 (14.07–20.27) |
| Kinda | 320 | 31 | 9.64 (7.67–12.34) |
| Kiruna | 657 | 65 | 9.90 (7.83–13.53) |
| Klippan | 484 | 72 | 14.81 (14.19–15.31) |
| Knivsta | 849 | 113 | 13.35 (11.39–15.63) |
| Köping | 710 | 141 | 19.92 (17.38–23.21) |
| Kramfors | 503 | 60 | 11.92 (9.96–13.24) |
| Kristianstad | 2560 | 376 | 14.69 (13.29–16.03) |
| Kristinehamn | 642 | 71 | 11.14 (7.17–13.57) |
| Krokom | 642 | 82 | 12.77 (10.30–15.24) |
| Kumla | 749 | 67 | 8.97 (6.45–12.85) |
| Kungälv | 1561 | 164 | 10.49 (8.84–12.34) |
| Kungsbacka | 3315 | 258 | 7.80 (6.48–9.51) |
| Kungsör | 249 | 20 | 8.00 (6.19–9.73) |
| Laholm | 717 | 57 | 7.92 (6.78–9.26) |
| Landskrona | 1260 | 172 | 13.64 (12.07–14.8) |
| Laxå | 123 | 11 | 8.59 (6.20–11.59) |
| Lekeberg | 261 | 56 | 21.43 (13.47–26.12) |
| Leksand | 440 | 75 | 17.12 (14.20–20.51) |
| Lerum | 1758 | 188 | 10.69 (9.35–12.36) |
| Lessebo | 246 | 32 | 12.9 (10.41–17.08) |
| Lidingö | 1883 | 245 | 12.99 (10.01–17.47) |
| Lidköping | 1230 | 171 | 13.91 (10.50–18.8) |
| Lilla Edet | 408 | 26 | 6.48 (5.20–8.62) |
| Lindesberg | 637 | 63 | 9.86 (7.02–13.48) |
| Linköping | 4805 | 517 | 10.75 (8.01–12.74) |
| Ljungby | 885 | 95 | 10.71 (9.92–11.87) |
| Ljusdal | 583 | 60 | 10.25 (8.02–12.71) |
| Ljusnarsberg | 97 | 8 | 7.89 (6.01–9.87) |
| Lomma | 1084 | 182 | 16.84 (15.39–18.8) |
| Ludvika | 714 | 108 | 15.15 (11.7–19.61) |
| Luleå | 2285 | 285 | 12.49 (10.09–14.79) |
| Lund | 3631 | 478 | 13.16 (11.11–15.29) |
| Lycksele | 357 | 38 | 10.62 (8.69–12.68) |
| Lysekil | 390 | 32 | 8.30 (7.27–10.02) |
| Malå | 98 | 21 | 21.6 (16.75–27.65) |
| Malmö | 9133 | 1304 | 14.28 (13.06–15.49) |
| Malung–Sälen | 273 | 37 | 13.69 (10.64–16.57) |
| Mariestad | 647 | 49 | 7.54 (6.20–9.91) |
| Mark | 1201 | 121 | 10.04 (8.51–11.14) |
| Markaryd | 275 | 41 | 14.91 (13.05–16.74) |
| Mellerud | 210 | 44 | 21.08 (16.33–25) |
| Mjölby | 906 | 141 | 15.53 (12.08–18.57) |
| Mölndal | 2360 | 222 | 9.42 (8.36–10.19) |
| Mönsterås | 344 | 48 | 13.84 (9.84–22.25) |
| Mora | 617 | 131 | 21.23 (17.89–25.25) |
| Mörbylånga | 472 | 36 | 7.61 (4.60–12.64) |
| Motala | 1302 | 130 | 10 (8.81–11.67) |
| Mullsjö | 283 | 27 | 9.42 (6.16–13.79) |
| Munkedal | 302 | 30 | 9.99 (9.26–10.73) |
| Munkfors | 101 | 24 | 23.50 (17.00–31.00) |
| Nacka | 4178 | 482 | 11.54 (9.15–13.83) |
| Nässjö | 850 | 75 | 8.87 (6.31–10.98) |
| Nora | 332 | 67 | 20.29 (16.7–24.6) |
| Norberg | 149 | 14 | 9.58 (7.46–11.88) |
| Nordanstig | 270 | 48 | 17.8 (15.25–19.64) |
| Nordmaling | 198 | 22 | 11.16 (9.79–12.63) |
| Norrköping | 4344 | 296 | 6.8 (6.12–7.70) |
| Norrtälje | 1614 | 202 | 12.52 (9.04–14.35) |
| Norsjö | 131 | 19 | 14.51 (10.68–20.16) |
| Nybro | 531 | 127 | 23.96 (17.55–29.74) |
| Nyköping | 1757 | 248 | 14.12 (12.82–15.93) |
| Nykvarn | 415 | 41 | 9.84 (5.66–13.68) |
| Nynäshamn | 820 | 134 | 16.32 (14.06–18.71) |
| Ockelbo | 166 | 16 | 9.76 (6.88–13.4) |
| Öckerö | 501 | 53 | 10.55 (9.24–12.26) |
| Ödeshög | 151 | 12 | 7.63 (5.41–10.01) |
| Olofström | 333 | 58 | 17.33 (11.99–20.83) |
| Örebro | 4523 | 545 | 12.04 (10.04–14.5) |
| Örkelljunga | 298 | 20 | 6.86 (5.46–8.07) |
| Örnsköldsvik | 1751 | 300 | 17.14 (14.64–19.28) |
| Orsa | 174 | 30 | 17.25 (10.46–20.38) |
| Orust | 428 | 36 | 8.43 (7.15–9.64) |
| Osby | 397 | 46 | 11.67 (10.08–13.56) |
| Oskarshamn | 802 | 70 | 8.78 (6.85–10.66) |
| Österåker | 1803 | 322 | 17.85 (14.03–21.35) |
| Östersund | 1973 | 178 | 9.03 (6.83–11.9) |
| Östhammar | 667 | 90 | 13.49 (11.23–16.04) |
| Östra Göinge | 412 | 48 | 11.75 (10.45–12.91) |
| Ovanåker | 301 | 44 | 14.5 (11.29–17.05) |
| Överkalix | 59 | 8 | 13.07 (10.59–16.07) |
| Övertorneå | 106 | 16 | 15.35 (12.66–18.23) |
| Oxelösund | 311 | 28 | 9.02 (6.79–11.13) |
| Pajala | 165 | 31 | 18.50 (13.76–23.69) |
| Partille | 1381 | 139 | 10.08 (8.73–11.81) |
| Perstorp | 184 | 25 | 13.43 (10.55–15.55) |
| Piteå | 1261 | 222 | 17.58 (14.37–22.22) |
| Ragunda | 150 | 20 | 13.12 (11.07–15.19) |
| Rättvik | 285 | 56 | 19.51 (14.12–25.13) |
| Robertsfors | 200 | 25 | 12.50 (10.39–15.21) |
| Ronneby | 808 | 118 | 14.64 (11.48–17.56) |
| Säffle | 414 | 26 | 6.18 (5.14–7.21) |
| Sala | 649 | 92 | 14.16 (13.09–16.81) |
| Salem | 741 | 90 | 12.12 (8.84–15.75) |
| Sandviken | 1111 | 96 | 8.63 (6.64–10.70) |
| Säter | 338 | 48 | 14.05 (11.18–17.46) |
| Sävsjö | 329 | 38 | 11.43 (9.2–14.06) |
| Sigtuna | 1529 | 320 | 20.93 (17.35–26.07) |
| Simrishamn | 471 | 53 | 11.35 (9.38–12.77) |
| Sjöbo | 564 | 60 | 10.56 (8.66–12.7) |
| Skara | 553 | 55 | 9.99 (8.73–12.20) |
| Skellefteå | 2145 | 239 | 11.15 (9.06–14.31) |
| Skinnskatteberg | 129 | 15 | 11.92 (11.31–13.03) |
| Skövde | 1592 | 132 | 8.29 (6.35–11.07) |
| Skurup | 550 | 66 | 11.96 (10.33–13.83) |
| Smedjebacken | 294 | 43 | 14.71 (12.60–17.53) |
| Söderhamn | 646 | 90 | 13.87 (11.00–16.46) |
| Söderköping | 451 | 69 | 15.40 (12.79–19.07) |
| Södertälje | 2970 | 329 | 11.07 (8.15–14.52) |
| Sollefteå | 543 | 98 | 18.07 (15.41–21.67) |
| Sollentuna | 2929 | 335 | 11.45 (9.49–15.10) |
| Solna | 1700 | 213 | 12.51 (10.04–15.64) |
| Sölvesborg | 479 | 76 | 15.89 (13.35–18.65) |
| Sorsele | 57 | 5 | 9.37 (6.93–11.95) |
| Sotenäs | 214 | 20 | 9.14 (8.05–10.12) |
| Staffanstorp | 1008 | 124 | 12.32 (9.73–16.25) |
| Stenungsund | 948 | 112 | 11.76 (9.76–14.18) |
| Stockholm | 27,098 | 2952 | 10.89 (8–13.42) |
| Storfors | 89 | 9 | 9.62 (6.59–12.99) |
| Storuman | 161 | 41 | 25.19 (20.69–29.07) |
| Strängnäs | 1254 | 133 | 10.63 (8.14–13.35) |
| Strömstad | 337 | 37 | 10.91 (8.09–14.24) |
| Strömsund | 294 | 48 | 16.25 (12.84–18.99) |
| Sundbyberg | 1196 | 123 | 10.31 (8.12–13.45) |
| Sundsvall | 3257 | 288 | 8.85 (6.56–11.23) |
| Sunne | 361 | 34 | 9.33 (5.95–14.45) |
| Surahammar | 318 | 46 | 14.55 (12.67–16.69) |
| Svalöv | 450 | 52 | 11.45 (10.06–12.95) |
| Svedala | 884 | 131 | 14.85 (12.08–16.89) |
| Svenljunga | 291 | 37 | 12.79 (10.94–14.72) |
| Täby | 2877 | 564 | 19.62 (14.12–24.79) |
| Tanum | 337 | 39 | 11.48 (10.47–12.49) |
| Tibro | 347 | 34 | 9.75 (8.06–12.1) |
| Tidaholm | 355 | 41 | 11.60 (8.71–14.28) |
| Tierp | 602 | 69 | 11.40 (9.16–13.63) |
| Timrå | 620 | 86 | 13.81 (12.36–15.28) |
| Tingsryd | 323 | 32 | 9.94 (7.5–13.23) |
| Tjörn | 500 | 44 | 8.71 (8.1–9.43) |
| Tomelilla | 388 | 44 | 11.30 (8.24–14.47) |
| Töreboda | 245 | 19 | 7.67 (5.44–10.51) |
| Torsås | 196 | 34 | 17.41 (12.89–23.15) |
| Torsby | 288 | 25 | 8.75 (5.63–12.49) |
| Tranås | 564 | 104 | 18.42 (15.31–21.95) |
| Tranemo | 338 | 32 | 9.50 (8.36–10.88) |
| Trelleborg | 1495 | 207 | 13.86 (12.54–15.48) |
| Trollhättan | 1813 | 147 | 8.09 (5.85–11.02) |
| Trosa | 449 | 79 | 17.62 (13.26–23.29) |
| Tyresö | 1914 | 287 | 15.01 (10.13–19.1) |
| Uddevalla | 1651 | 181 | 10.96 (8.46–14.75) |
| Ulricehamn | 727 | 93 | 12.79 (10.49–16.49) |
| Umeå | 3739 | 440 | 11.76 (8.97–15.11) |
| Upplands Väsby | 1448 | 191 | 13.20 (10.47–16.1) |
| Upplands–Bro | 1016 | 241 | 23.73 (18.51–30.88) |
| Uppsala | 6536 | 417 | 6.37 (4.68–8.62) |
| Uppvidinge | 243 | 35 | 14.50 (9.7–21.43) |
| Vadstena | 199 | 30 | 14.90 (11.46–18.18) |
| Vaggeryd | 501 | 93 | 18.57 (15.41–22.5) |
| Valdemarsvik | 192 | 15 | 8.03 (6.32–9.85) |
| Vallentuna | 1402 | 174 | 12.44 (11.08–14.31) |
| Vänersborg | 1163 | 76 | 6.55 (4.90–8.20) |
| Vännäs | 268 | 52 | 19.5 (14.45–24.95) |
| Vansbro | 193 | 39 | 20.11 (15.68–23.72) |
| Vara | 474 | 56 | 11.83 (9.88–13.74) |
| Varberg | 1978 | 186 | 9.42 (8.19–11.18) |
| Vårgårda | 399 | 57 | 14.17 (12.09–16.6) |
| Värmdö | 1809 | 199 | 10.99 (9.32–13.17) |
| Värnamo | 1073 | 94 | 8.78 (7.98–9.9) |
| Västerås | 4559 | 740 | 16.24 (14.38–17.61) |
| Västervik | 1020 | 165 | 16.17 (12.10–19.30) |
| Vaxholm | 541 | 86 | 15.93 (12.18–19.32) |
| Växjö | 2818 | 429 | 15.22 (12.85–17.72) |
| Vellinge | 1417 | 139 | 9.81 (8.24–11.73) |
| Vetlanda | 760 | 73 | 9.55 (7.78–11.47) |
| Vilhelmina | 230 | 32 | 14.08 (12.54–15.54) |
| Vimmerby | 431 | 40 | 9.31 (7.80–10.79) |
| Vindeln | 160 | 14 | 8.78 (7.41–10.83) |
| Vingåker | 232 | 28 | 12.25 (10.34–14.82) |
| Ydre | 82 | 9 | 10.81 (9.38–13.32) |
| Ystad | 872 | 126 | 14.44 (11.12–17.70) |
